# Poly[μ_3_-aqua-aqua-μ_5_-(4-nitro­benzoato)-caesium]

**DOI:** 10.1107/S1600536813030638

**Published:** 2013-11-16

**Authors:** Graham Smith

**Affiliations:** a Science and Engineering Faculty, Queensland University of Technology, GPO Box 2434, Brisbane, Queensland 4001, Australia

## Abstract

In the structure of the title complex, [Cs(C_7_H_4_NO_2_)(H_2_O)_2_]_*n*_, the caesium salt of 4-nitro­benzoic acid, the irregular CsO_9_ coordination sphere comprises three bridging nitro O-atom donors, a bidentate carboxyl­ate *O*,*O*′-chelate inter­action, a triple-bridging water mol­ecule and a monodentate water mol­ecule. A three-dimensional framework polymer is generated, within which there are water–carboxyl­ate O—H⋯O and water–water O—H⋯O hydrogen-bonding inter­actions.

## Related literature

For structures of alkali metal salts of 4-nitro­benzoic acid, see: Turowska-Tyrk *et al.* (1988 ▶) (Na); Srivastava & Speakman (1961 ▶) (K). For the structures of Na, K and Cs complexes with 4-nitro­anthranilic acid, see: Smith & Wermuth (2011 ▶); Smith (2013 ▶). For the structures of the 4-nitro­benzoic acid polymorphs, see: Groth (1980 ▶); Tonogaki *et al.* (1993 ▶); Bolte (2009 ▶).
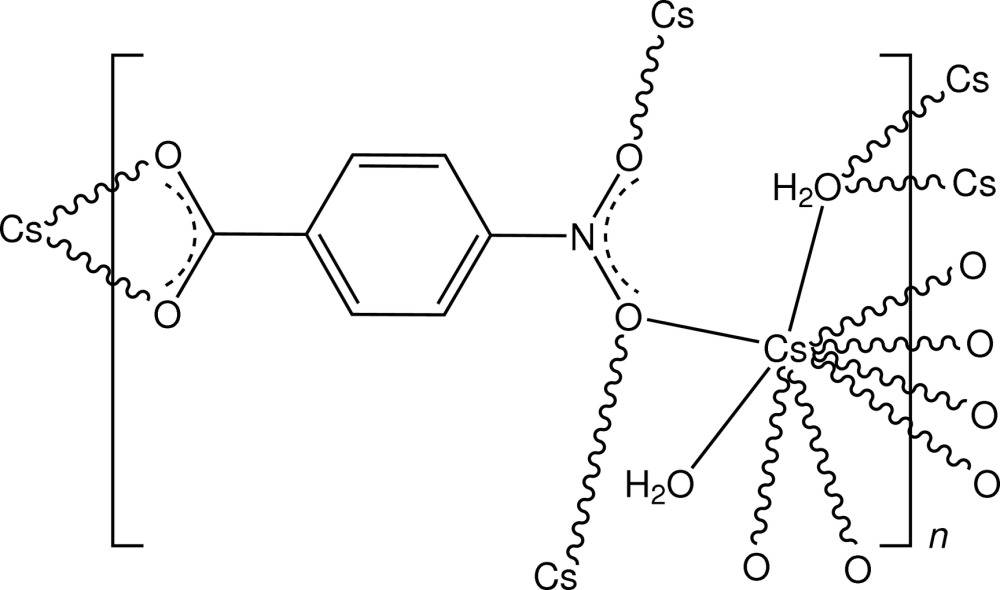


## Experimental

### 

#### Crystal data

[Cs(C_7_H_4_NO_2_)(H_2_O)_2_]
*M* *_r_* = 335.05
Monoclinic, 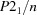

*a* = 6.0700 (3) Å
*b* = 7.1073 (4) Å
*c* = 24.2183 (13) Å
β = 94.035 (5)°
*V* = 1042.22 (10) Å^3^
*Z* = 4
Mo *K*α radiation
μ = 3.56 mm^−1^
*T* = 200 K
0.28 × 0.18 × 0.05 mm

#### Data collection

Oxford Diffraction Gemini-S CCD-detector diffractometer
Absorption correction: multi-scan (*CrysAlis PRO*; Agilent, 2012 ▶) *T*_min_ = 0.604, *T*_max_ = 0.980
6334 measured reflections
2057 independent reflections
1836 reflections with *I* > 2σ(*I*)
*R*_int_ = 0.036

#### Refinement

*R*[*F* ^2^ > 2σ(*F* ^2^)] = 0.032
*wR*(*F* ^2^) = 0.074
*S* = 1.15
2057 reflections
136 parameters
H-atom parameters constrained
Δρ_max_ = 0.56 e Å^−3^
Δρ_min_ = −0.67 e Å^−3^

### 

Data collection: *CrysAlis PRO* (Agilent, 2012 ▶); cell refinement: *CrysAlis PRO*; data reduction: *CrysAlis PRO*; program(s) used to solve structure: *SIR92* (Altomare *et al.*, 1993 ▶); program(s) used to refine structure: *SHELXL97* (Sheldrick, 2008 ▶) within *WinGX* (Farrugia, 2012 ▶); molecular graphics: *PLATON* (Spek, 2009 ▶); software used to prepare material for publication: *PLATON*.

## Supplementary Material

Crystal structure: contains datablock(s) global, I. DOI: 10.1107/S1600536813030638/lh5666sup1.cif

Structure factors: contains datablock(s) I. DOI: 10.1107/S1600536813030638/lh5666Isup2.hkl

Supplementary material file. DOI: 10.1107/S1600536813030638/lh5666Isup3.cml

Additional supplementary materials:  crystallographic information; 3D view; checkCIF report

## Figures and Tables

**Table 1:** Selected bond lengths (Å)

Cs1—O1*W*	3.126 (3)
Cs1—O2*W*	3.253 (3)
Cs1—O41	3.244 (4)
Cs1—O2*W* ^i^	3.220 (3)
Cs1—O42^i^	3.248 (4)
Cs1—O11^ii^	3.215 (3)
Cs1—O12^ii^	3.338 (4)
Cs1—O2*W* ^iii^	3.047 (4)
Cs1—O41^iii^	3.310 (4)

Symmetry codes: (i) 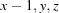; (ii) 
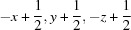
; (iii) 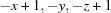.

**Table 2:** Hydrogen-bond geometry (Å, °)

*D*—H⋯*A*	*D*—H	H⋯*A*	*D*⋯*A*	*D*—H⋯*A*
O1*W*—H11*W*⋯O12^iv^	0.82	1.88	2.694 (5)	174
O1*W*—H12*W*⋯O11^v^	0.93	1.81	2.728 (4)	173
O2*W*—H21*W*⋯O1*W* ^vi^	0.79	1.99	2.749 (5)	162
O2*W*—H22*W*⋯O11^iv^	0.84	1.91	2.753 (5)	174

Symmetry codes: (iv) 
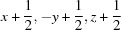
; (v) 
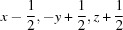
; (vi) 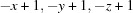.

**Fig. 1.**
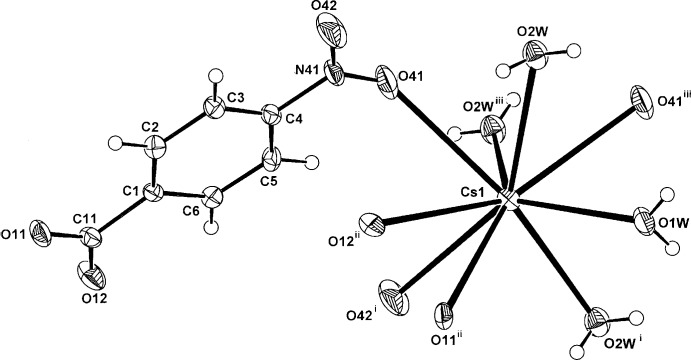
The molecular configuration and atom-numbering scheme for the coordination polyhedron of title complex, with non-H atoms drawn as 40% probability displacement ellipsoids. For symmetry codes: see Table 1.

**Fig. 2.**
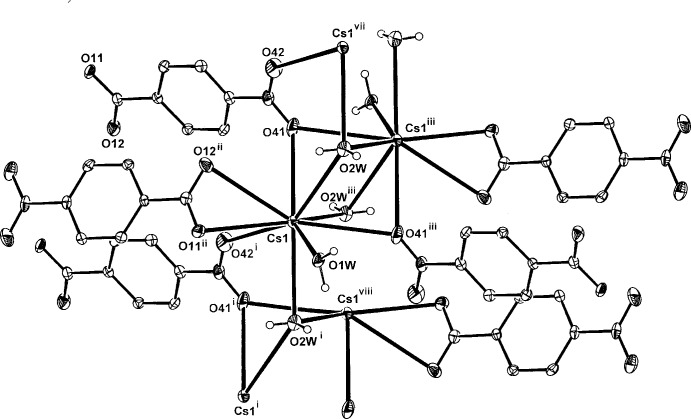
A partial expansion of the CsO9 coordination sphere in the polymeric structure. For symmetry codes: (vii) x + 1, y, z; (viii) -x, -y, -z + 1. For other symmetry codes, see Fig. 1 and Table 1.

**Fig. 3.**
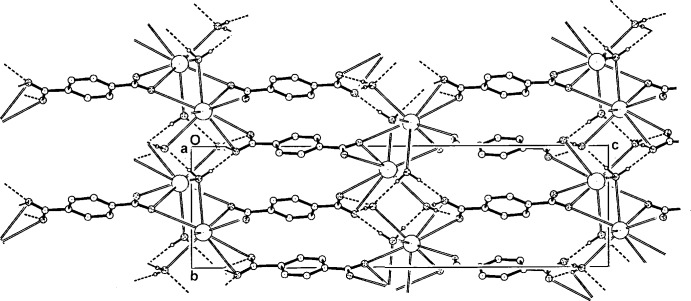
The packing of the structure in the unit cell viewed along a. Hydrogen-bonding associations are shown as dashed lines.

## supplementary crystallographic information

### 1. Comment

4-Nitrobenzoic acid (PNBA) has proved to be a useful ligand for the preparation of metal complexes, which are mainly monomeric but rarely involve the nitro group in coordination. With the known alkali metal salts of PNBA, the sodium salt (a trihydrate) (Turowska-Tyrk *et al.*, 1988) and the potassium salt (a 1:1 salt-acid adduct) (Srivastava & Speakman, 1961), coordination polymeric structures are formed, but the structures of the rubidium and caesium salts have not been reported. The reaction of 4-nitrobenzoic acid with caesium hydroxide in aqueous ethanol afforded good crystals of the title Cs complex, [Cs(C_7_H_4_NO_2_)(H_2_O)_2_]_n_ and the structure is reported herein.

In this structure (Fig. 1), the irregular CsO_9_ coordinate polyhedron comprises a bidentate carboxylate *O,O'*-chelate interaction, three O-donors from an *O,O'*-bridging nitro group, three O donors from a triple-bridging water molecule (O2*W*) and a monodentate water molecule (O1*W*) [Cs—O, 3.047 (4)–3.338 (4) Å] (Table 1). The bridging extensions in the two-dimensional sheet substructures which extend along the (0 0 1) plane include a centrosymmetric water–carboxyl quadruple cage (Fig. 2) (Cs···Cs^iii^ = 4.2610 (6) Å] [for symmetry code (iii), see Table 2]. The *p*-related carboxyl and nitro substituent groups of the PNBA ligand link the sheets across *c*, and generate an overall a three-dimensional coordination polymer (Fig. 3). This type of structure extension through the *p*-related benzoate carboxyl and nitro functional groups is similar to that found in other alkali metal complexes with the 4-nitroanthranilate salts of sodium (a dihydrate) and potassium (a monohydrate) (Smith, 2013), and caesium (a monohydrate) (Smith & Wermuth, 2011).

The crystal structure of the title complex polymer is stabilized by intra-sheet water O—H···O_carboxyl_ and O—H···O_water_ hydrogen-bonding interactions (Table 2). No inter-ring π–π interactions are present [minimun ring centroid separation 4.643 (2) Å]. The PABA ligand in the complex is essentially planar [torsion angles C2—C1—C11—O12 = 177.9 (4)° (carboxyl) and C3—C4—N41—O41 = 177.5 (4)° (nitro)]. This conformation is similar to that found in both monoclinic polymorphs of the parent acid [Tonogaki *et al.*, 1993; Groth, 1980; Bolte, 2009].

### 2. Experimental

The title compound was synthesized by heating together for 10 minutes, 0.5 mmol of 4-nitrobenzoic acid and 0.5 mmol of CsOH in 15 ml of 10% ethanol–water. Partial room temperature evaporation of the solution gave colourless elongated crystal plates of the title complex from which a specimen was cleaved for the X-ray analysis.

### 3. Refinement

Carbon-bound hydrogen atoms were placed in calculated positions [C—H = 0.95 Å] and allowed to ride in the refinement, with *U*_iso_(H) = 1.2*U*_eq_(C). Hydrogen atoms of the coordinated water molecule were located in a difference Fourier map but were subsequently allowed to ride, with *U*_iso_(H) = 1.5*U*_eq_(O).

### Crystal data

**Table:**

[Cs(C_7_H_4_NO_2_)(H_2_O)_2_]	*F*(000) = 640
*M**_r_* = 335.05	*D*_x_ = 2.135 Mg m^−^^3^
Monoclinic, *P*2_1_/*n*	Mo *K*α radiation, λ = 0.71073 Å
Hall symbol: -P 2yn	Cell parameters from 2366 reflections
*a* = 6.0700 (3) Å	θ = 3.5–28.1°
*b* = 7.1073 (4) Å	µ = 3.56 mm^−^^1^
*c* = 24.2183 (13) Å	*T* = 200 K
β = 94.035 (5)°	Plate, colourless
*V* = 1042.22 (10) Å^3^	0.28 × 0.18 × 0.05 mm
*Z* = 4	

### Data collection

**Table:**

Oxford Diffraction Gemini-S CCD-detector diffractometer	2057 independent reflections
Radiation source: Enhance (Mo) X-ray source	1836 reflections with *I* > 2σ(*I*)
Graphite monochromator	*R*_int_ = 0.036
Detector resolution: 16.077 pixels mm^-1^	θ_max_ = 26.0°, θ_min_ = 3.3°
ω scans	*h* = −7→7
Absorption correction: multi-scan (*CrysAlis PRO*; Agilent, 2012)	*k* = −8→8
*T*_min_ = 0.604, *T*_max_ = 0.980	*l* = −22→29
6334 measured reflections	

### Refinement

**Table:**

Refinement on *F*^2^	Primary atom site location: structure-invariant direct methods
Least-squares matrix: full	Secondary atom site location: difference Fourier map
*R*[*F*^2^ > 2σ(*F*^2^)] = 0.032	Hydrogen site location: inferred from neighbouring sites
*wR*(*F*^2^) = 0.074	H-atom parameters constrained
*S* = 1.15	*w* = 1/[σ^2^(*F*_o_^2^) + (0.0285*P*)^2^ + 0.878*P*] where *P* = (*F*_o_^2^ + 2*F*_c_^2^)/3
2057 reflections	(Δ/σ)_max_ = 0.001
136 parameters	Δρ_max_ = 0.56 e Å^−^^3^
0 restraints	Δρ_min_ = −0.67 e Å^−^^3^

### Special details

**Table:**

Geometry. Bond distances, angles *etc*. have been calculated using the rounded fractional coordinates. All su's are estimated from the variances of the (full) variance-covariance matrix. The cell e.s.d.'s are taken into account in the estimation of distances, angles and torsion angles
Refinement. Refinement of *F*^2^ against ALL reflections. The weighted *R*-factor *wR* and goodness of fit *S* are based on *F*^2^, conventional *R*-factors *R* are based on *F*, with *F* set to zero for negative *F*^2^. The threshold expression of *F*^2^ > σ(*F*^2^) is used only for calculating *R*-factors(gt) *etc*. and is not relevant to the choice of reflections for refinement. *R*-factors based on *F*^2^ are statistically about twice as large as those based on *F*, and *R*- factors based on ALL data will be even larger.

### Fractional atomic coordinates and isotropic or equivalent isotropic displacement parameters (Å^2^)

**Table:**

	*x*	*y*	*z*	*U*_iso_*/*U*_eq_	
Cs1	0.25378 (4)	0.19824 (4)	0.47189 (1)	0.0259 (1)	
O1W	0.2785 (5)	0.5049 (5)	0.56462 (14)	0.0354 (11)	
O2W	0.7695 (5)	0.2283 (5)	0.51753 (15)	0.0347 (11)	
O11	0.3802 (5)	0.0475 (5)	0.10793 (14)	0.0333 (11)	
O12	0.0968 (5)	−0.0945 (5)	0.14338 (15)	0.0400 (11)	
O41	0.6184 (7)	−0.0192 (5)	0.40379 (16)	0.0467 (14)	
O42	0.9117 (7)	0.0828 (7)	0.36824 (17)	0.0576 (16)	
N41	0.7213 (7)	0.0261 (6)	0.36407 (17)	0.0311 (12)	
C1	0.4023 (7)	−0.0137 (5)	0.20429 (19)	0.0189 (11)	
C2	0.6135 (7)	0.0617 (6)	0.2120 (2)	0.0236 (14)	
C3	0.7195 (7)	0.0745 (6)	0.26440 (19)	0.0235 (14)	
C4	0.6093 (7)	0.0113 (6)	0.30818 (19)	0.0229 (14)	
C5	0.4009 (7)	−0.0646 (6)	0.30250 (19)	0.0250 (14)	
C6	0.2985 (7)	−0.0777 (6)	0.25023 (19)	0.0223 (14)	
C11	0.2830 (7)	−0.0201 (6)	0.1477 (2)	0.0252 (14)	
H2	0.68620	0.10490	0.18090	0.0280*	
H3	0.86400	0.12560	0.26980	0.0280*	
H5	0.32940	−0.10700	0.33390	0.0300*	
H6	0.15490	−0.13100	0.24530	0.0270*	
H11W	0.37890	0.53710	0.58680	0.0530*	
H12W	0.14960	0.48830	0.58230	0.0530*	
H21W	0.72670	0.30190	0.49470	0.0520*	
H22W	0.79800	0.29200	0.54650	0.0520*	

### Atomic displacement parameters (Å^2^)

**Table:**

	*U*^11^	*U*^22^	*U*^33^	*U*^12^	*U*^13^	*U*^23^
Cs1	0.0259 (2)	0.0272 (2)	0.0240 (2)	0.0000 (1)	−0.0015 (1)	0.0013 (1)
O1W	0.0348 (18)	0.052 (2)	0.0189 (19)	−0.0046 (17)	−0.0014 (14)	0.0006 (16)
O2W	0.044 (2)	0.0296 (17)	0.030 (2)	−0.0030 (15)	−0.0003 (16)	−0.0028 (15)
O11	0.0334 (18)	0.049 (2)	0.0173 (19)	−0.0022 (16)	0.0010 (14)	0.0089 (16)
O12	0.0335 (19)	0.055 (2)	0.030 (2)	−0.0104 (18)	−0.0092 (15)	0.0061 (18)
O41	0.065 (3)	0.056 (2)	0.018 (2)	0.002 (2)	−0.0040 (18)	0.0036 (18)
O42	0.049 (2)	0.085 (3)	0.036 (3)	−0.015 (2)	−0.0171 (19)	−0.007 (2)
N41	0.042 (2)	0.033 (2)	0.017 (2)	0.0051 (19)	−0.0076 (19)	−0.0057 (18)
C1	0.020 (2)	0.0163 (19)	0.020 (2)	0.0015 (17)	−0.0021 (18)	−0.0017 (18)
C2	0.027 (2)	0.021 (2)	0.023 (3)	−0.0022 (18)	0.0028 (19)	0.0012 (19)
C3	0.023 (2)	0.024 (2)	0.023 (3)	−0.0029 (19)	−0.0008 (19)	−0.0037 (19)
C4	0.028 (2)	0.020 (2)	0.020 (3)	0.0046 (19)	−0.0041 (19)	−0.0051 (18)
C5	0.034 (2)	0.024 (2)	0.018 (3)	0.001 (2)	0.008 (2)	0.0026 (19)
C6	0.023 (2)	0.021 (2)	0.023 (3)	−0.0010 (18)	0.0028 (18)	0.0009 (19)
C11	0.028 (2)	0.025 (2)	0.022 (3)	0.005 (2)	−0.003 (2)	0.002 (2)

### Geometric parameters (Å, º)

**Table:**

Cs1—O1W	3.126 (3)	O2W—H21W	0.7900
Cs1—O2W	3.253 (3)	O2W—H22W	0.8400
Cs1—O41	3.244 (4)	N41—C4	1.475 (6)
Cs1—O2W^i^	3.220 (3)	C1—C2	1.390 (6)
Cs1—O42^i^	3.248 (4)	C1—C6	1.393 (6)
Cs1—O11^ii^	3.215 (3)	C1—C11	1.505 (7)
Cs1—O12^ii^	3.338 (4)	C2—C3	1.385 (7)
Cs1—O2W^iii^	3.047 (4)	C3—C4	1.369 (6)
Cs1—O41^iii^	3.310 (4)	C4—C5	1.373 (6)
O11—C11	1.260 (6)	C5—C6	1.374 (6)
O12—C11	1.246 (5)	C2—H2	0.9500
O41—N41	1.226 (6)	C3—H3	0.9500
O42—N41	1.221 (6)	C5—H5	0.9500
O1W—H11W	0.8200	C6—H6	0.9500
O1W—H12W	0.9300		
			
O1W—Cs1—O2W	73.36 (8)	Cs1^v^—O12—C11	87.7 (3)
O1W—Cs1—O41	134.24 (9)	Cs1—O41—N41	132.6 (3)
O1W—Cs1—O2W^i^	72.89 (8)	Cs1—O41—Cs1^iii^	81.10 (9)
O1W—Cs1—O42^i^	136.65 (10)	Cs1^iii^—O41—N41	135.6 (3)
O1W—Cs1—O11^ii^	83.74 (9)	Cs1^iv^—O42—N41	134.2 (3)
O1W—Cs1—O12^ii^	106.92 (9)	H11W—O1W—H12W	110.00
O1W—Cs1—O2W^iii^	129.32 (9)	Cs1—O1W—H11W	132.00
O1W—Cs1—O41^iii^	67.55 (9)	Cs1—O1W—H12W	104.00
O2W—Cs1—O41	61.92 (9)	Cs1^iv^—O2W—H21W	94.00
O2W—Cs1—O2W^i^	139.38 (9)	Cs1—O2W—H21W	63.00
O2W—Cs1—O42^i^	145.77 (10)	Cs1—O2W—H22W	117.00
O2W—Cs1—O11^ii^	110.50 (8)	H21W—O2W—H22W	105.00
O2W—Cs1—O12^ii^	86.77 (8)	Cs1^iii^—O2W—H22W	118.00
O2W—Cs1—O2W^iii^	94.93 (9)	Cs1^iii^—O2W—H21W	135.00
O2W—Cs1—O41^iii^	63.75 (10)	Cs1^iv^—O2W—H22W	101.00
O2W^i^—Cs1—O41	151.44 (9)	O41—N41—O42	123.6 (4)
O41—Cs1—O42^i^	84.76 (11)	O42—N41—C4	118.2 (4)
O11^ii^—Cs1—O41	102.41 (9)	O41—N41—C4	118.3 (4)
O12^ii^—Cs1—O41	63.24 (9)	C2—C1—C11	120.9 (4)
O2W^iii^—Cs1—O41	66.80 (9)	C2—C1—C6	118.9 (4)
O41—Cs1—O41^iii^	98.90 (10)	C6—C1—C11	120.1 (4)
O2W^i^—Cs1—O42^i^	74.49 (10)	C1—C2—C3	120.9 (4)
O2W^i^—Cs1—O11^ii^	87.52 (8)	C2—C3—C4	117.9 (4)
O2W^i^—Cs1—O12^ii^	124.49 (8)	C3—C4—C5	123.3 (4)
O2W^i^—Cs1—O2W^iii^	89.33 (9)	N41—C4—C3	118.0 (4)
O2W^i^—Cs1—O41^iii^	82.79 (10)	N41—C4—C5	118.8 (4)
O11^ii^—Cs1—O42^i^	67.02 (11)	C4—C5—C6	118.2 (4)
O12^ii^—Cs1—O42^i^	70.23 (10)	C1—C6—C5	120.9 (4)
O2W^iii^—Cs1—O42^i^	77.45 (11)	O11—C11—O12	124.7 (4)
O41^iii^—Cs1—O42^i^	134.58 (11)	O11—C11—C1	117.6 (4)
O11^ii^—Cs1—O12^ii^	39.52 (8)	O12—C11—C1	117.7 (4)
O2W^iii^—Cs1—O11^ii^	143.86 (9)	C1—C2—H2	120.00
O11^ii^—Cs1—O41^iii^	151.25 (9)	C3—C2—H2	120.00
O2W^iii^—Cs1—O12^ii^	121.77 (9)	C2—C3—H3	121.00
O12^ii^—Cs1—O41^iii^	150.49 (9)	C4—C3—H3	121.00
O2W^iii^—Cs1—O41^iii^	63.27 (9)	C4—C5—H5	121.00
Cs1—O2W—Cs1^iv^	139.38 (12)	C6—C5—H5	121.00
Cs1—O2W—Cs1^iii^	85.07 (8)	C1—C6—H6	119.00
Cs1^iv^—O2W—Cs1^iii^	90.67 (9)	C5—C6—H6	120.00
Cs1^v^—O11—C11	93.0 (3)		
			
O1W—Cs1—O2W—Cs1^iv^	−145.1 (2)	O1W—Cs1—O12^ii^—C11^ii^	−76.3 (3)
O1W—Cs1—O2W—Cs1^iii^	129.64 (10)	O2W—Cs1—O12^ii^—C11^ii^	−147.8 (3)
O41—Cs1—O2W—Cs1^iv^	24.89 (16)	O41—Cs1—O12^ii^—C11^ii^	152.2 (3)
O41—Cs1—O2W—Cs1^iii^	−60.38 (9)	O1W—Cs1—O2W^iii^—Cs1^iii^	−72.49 (11)
O2W^i^—Cs1—O2W—Cs1^iv^	−179.98 (15)	O2W—Cs1—O2W^iii^—Cs1^iii^	−0.03 (11)
O2W^i^—Cs1—O2W—Cs1^iii^	94.74 (13)	O41—Cs1—O2W^iii^—Cs1^iii^	56.56 (9)
O42^i^—Cs1—O2W—Cs1^iv^	10.4 (3)	O1W—Cs1—O41^iii^—Cs1^iii^	−134.37 (10)
O42^i^—Cs1—O2W—Cs1^iii^	−74.9 (2)	O1W—Cs1—O41^iii^—N41^iii^	80.5 (4)
O11^ii^—Cs1—O2W—Cs1^iv^	−68.59 (19)	O2W—Cs1—O41^iii^—Cs1^iii^	−52.52 (9)
O11^ii^—Cs1—O2W—Cs1^iii^	−153.85 (8)	O2W—Cs1—O41^iii^—N41^iii^	162.4 (4)
O12^ii^—Cs1—O2W—Cs1^iv^	−36.36 (18)	O41—Cs1—O41^iii^—Cs1^iii^	0.00 (10)
O12^ii^—Cs1—O2W—Cs1^iii^	−121.63 (9)	O41—Cs1—O41^iii^—N41^iii^	−145.1 (4)
O2W^iii^—Cs1—O2W—Cs1^iv^	85.26 (18)	Cs1^v^—O11—C11—C1	135.5 (3)
O2W^iii^—Cs1—O2W—Cs1^iii^	0.00 (9)	Cs1^v^—O11—C11—O12	−43.3 (5)
O41^iii^—Cs1—O2W—Cs1^iv^	142.2 (2)	Cs1^v^—O12—C11—O11	41.3 (4)
O41^iii^—Cs1—O2W—Cs1^iii^	56.92 (9)	Cs1^v^—O12—C11—C1	−137.5 (3)
O1W—Cs1—O41—N41	−79.8 (4)	Cs1—O41—N41—O42	99.6 (6)
O1W—Cs1—O41—Cs1^iii^	67.23 (13)	Cs1—O41—N41—C4	−80.5 (5)
O2W—Cs1—O41—N41	−93.3 (4)	Cs1^iii^—O41—N41—O42	−30.2 (7)
O2W—Cs1—O41—Cs1^iii^	53.78 (9)	Cs1^iii^—O41—N41—C4	149.7 (3)
O2W^i^—Cs1—O41—N41	121.7 (4)	Cs1^iv^—O42—N41—O41	−7.8 (8)
O2W^i^—Cs1—O41—Cs1^iii^	−91.25 (19)	Cs1^iv^—O42—N41—C4	172.3 (3)
O42^i^—Cs1—O41—N41	78.6 (4)	O41—N41—C4—C5	−4.5 (6)
O42^i^—Cs1—O41—Cs1^iii^	−134.36 (10)	O41—N41—C4—C3	175.5 (4)
O11^ii^—Cs1—O41—N41	13.5 (4)	O42—N41—C4—C5	175.5 (4)
O11^ii^—Cs1—O41—Cs1^iii^	160.58 (7)	O42—N41—C4—C3	−4.5 (6)
O12^ii^—Cs1—O41—N41	8.1 (4)	C11—C1—C2—C3	177.4 (4)
O12^ii^—Cs1—O41—Cs1^iii^	155.17 (11)	C2—C1—C6—C5	0.9 (6)
O2W^iii^—Cs1—O41—N41	157.2 (4)	C6—C1—C2—C3	−0.4 (6)
O2W^iii^—Cs1—O41—Cs1^iii^	−55.78 (8)	C6—C1—C11—O12	−4.4 (6)
O41^iii^—Cs1—O41—N41	−147.1 (4)	C11—C1—C6—C5	−176.9 (4)
O41^iii^—Cs1—O41—Cs1^iii^	0.00 (11)	C2—C1—C11—O11	−1.0 (6)
O1W—Cs1—O2W^i^—Cs1^i^	145.0 (2)	C2—C1—C11—O12	177.9 (4)
O2W—Cs1—O2W^i^—Cs1^i^	179.97 (17)	C6—C1—C11—O11	176.8 (4)
O41—Cs1—O2W^i^—Cs1^i^	−51.0 (3)	C1—C2—C3—C4	−0.3 (6)
O1W—Cs1—O42^i^—N41^i^	−63.5 (5)	C2—C3—C4—C5	0.4 (7)
O2W—Cs1—O42^i^—N41^i^	151.9 (4)	C2—C3—C4—N41	−179.6 (4)
O41—Cs1—O42^i^—N41^i^	139.1 (5)	C3—C4—C5—C6	0.1 (7)
O1W—Cs1—O11^ii^—C11^ii^	145.7 (3)	N41—C4—C5—C6	−180.0 (4)
O2W—Cs1—O11^ii^—C11^ii^	76.1 (3)	C4—C5—C6—C1	−0.7 (6)
O41—Cs1—O11^ii^—C11^ii^	11.7 (3)		

Symmetry codes: (i) *x*−1, *y*, *z*; (ii) −*x*+1/2, *y*+1/2, −*z*+1/2; (iii) −*x*+1, −*y*, −*z*+1; (iv) *x*+1, *y*, *z*; (v) −*x*+1/2, *y*−1/2, −*z*+1/2.

### Hydrogen-bond geometry (Å, º)

**Table:**

*D*—H···*A*	*D*—H	H···*A*	*D*···*A*	*D*—H···*A*
O1*W*—H11*W*···O12^vi^	0.82	1.88	2.694 (5)	174
O1*W*—H12*W*···O11^vii^	0.93	1.81	2.728 (4)	173
O2*W*—H21*W*···O1*W*^viii^	0.79	1.99	2.749 (5)	162
O2*W*—H22*W*···O11^vi^	0.84	1.91	2.753 (5)	174

Symmetry codes: (vi) *x*+1/2, −*y*+1/2, *z*+1/2; (vii) *x*−1/2, −*y*+1/2, *z*+1/2; (viii) −*x*+1, −*y*+1, −*z*+1.
